# Peptide-enriched hydrogel formulation for sensitive and damaged skin: from design to application testing

**DOI:** 10.1038/s41598-026-49562-4

**Published:** 2026-04-24

**Authors:** Deptuła Milena, Zawrzykraj Małgorzata, Sawicka Justyna, Dzierżyńska Maria, Skoniecka Aneta, Czerwiec Katarzyna, Kondej Karolina, Zieliński Jacek, Janus Łukasz, Rodziewicz-Motowidło Sylwia, Pikuła Michał

**Affiliations:** 1 Laboratory of Tissue Engineering and Regenerative Medicine, Division of Embryology, Medical University of Gdańsk, Gdańsk, Poland; 2 Division of Clinical Anatomy, Medical University of Gdańsk, Gdańsk, Poland; 3 Department of Biomedical Chemistry, Faculty of Chemistry, University of Gdańsk, Gdańsk, Poland; 4 Department of Plastic Surgery, Medical University of Gdańsk, Gdańsk, Poland; 5 Department of Surgical Oncology, Transplant Surgery and General Surgery, Medical University of Gdańsk, Gdańsk, Poland; 6 BioGel Sp. Z O.O., Szybowcowa 8a, 80-298 Gdańsk, Poland; 7 Department of Biochemistry, Gdańsk University of Physical Education and Sport, Gdańsk, Poland

**Keywords:** Biotechnology, Drug discovery, Health care, Medical research

## Abstract

Various factors such as infections, wounds, and comorbidities can disrupt the skin’s physiological function. Moreover, skin lesions can result from radiation therapy. We aimed to create a new cosmetic formulation with pro-regenerative properties that is specifically designed for patients with sustained skin damage, such as those who have undergone radiation therapy. In the first stage, NE1 and IM2 peptides were synthesized, and hydrogels were prepared. IM2 is a derivative of the IM peptide, while NE1 contains the bioactive GHK sequence. In the next stage physicochemical analyses were conducted, including the evaluation of peptide stability and the developed composition’s microbial purity and packaging compatibility. In addition, in vitro safety and activity assessments were implemented on human skin cells. In the final step, dermatological tests were conducted on the participants. The results indicate that the cosmetic composition is stable, possesses preservative properties, and is safe in both in vitro and in vivo studies. Peptide release studies show that within the first hours, approximately 75–80% of each peptide is released, ensuring a rapid onset of action. Analysis of cell migration indicate that both p407 and the designed composition stimulate migration of HaCaT keratinocytes in vitro. Dermatological tests did not show any irritant and sensitizing properties. Application analyses revealed that the designed composition effectively moisturizes and takes care of sensitive skin, alleviates redness and the effects of rough skin, eliminates the sensation of itching and the effects of skin tension, and soothes irritation. This formulation can be used for the daily care of sensitive, allergic, or irritated skin.

**Supplementary Information:**

The online version contains supplementary material available at 10.1038/s41598-026-49562-4.

## Introduction

The skin, the primary organ of the human body, provides mechanical protection, prevents water loss and regulates the immune and thermal functions^1^. The effective functioning of the epidermal barrier and the protection of the body from external factors are contingent on the preservation of skin homeostasis^2^. UV light, allergens, air pollution, and chemical or medical treatments, such as radiotherapy, are some factors that can damage the skin, impair barrier protection, and disrupt homeostasis^3^.

Depending on the type and stage of cancer, primary treatment often involves surgery, chemotherapy, radiation therapy, or immunotherapy^4^. In radiation therapy, oxygen-free radicals are used to damage DNA strands. However, this treatment can also harm nearby healthy cells and produce early and late lesions^5^. The early changes involve damage to the skin and the mucous membranes of the gastrointestinal tract and mouth. Late lesions may appear in brain cells, adipose tissue, kidneys, liver, intestinal wall, or muscles. They are primarily associated with processes such as atrophy, fibrosis, tissue necrosis, and vascular damage^6^.

Skin reactions are of the primary adverse effects of radiation therapy ranging from erythema and skin peeling to chronic ulcers and wounds. The main treatment of skin reactions—hyperbaric oxygen therapy, hydrogel dressings, creams, and ointments, and corticosteroids—are, however, not entirely effective. Alternative solutions are thus being pursued for post-radiation therapy skin care^7^.

There has been growing interest in peptide applications in medicine and cosmetology. Peptides are short, acid–based compounds that are either naturally occurring or synthesized. Owing to their anti-inflammatory, antimicrobial, and cell proliferating properties, they are useful for healing wounds^8^. Peptides are also used in cosmetics to improve skin cell proliferation, collagen synthesis, reduce inflammation and pigmentation spots or improve skin barrier function^9^. Cosmetic products intended for use on sensitive skin mainly contain synthetic peptides, based on pharmacologically actives sequences of natural molecules. The use of them allows overcoming the disadvantages of natural peptides like e.g. high molecular weight and containing allergenic moieties^10^. Both peptides used in the formulation prepared in this work are in line with the trends used in the development of cosmetics containing peptides. NE1 (Ac-Gly-His-Lys-Gly-Gly-Gly-Ala-Ala-Pro-Val-Gly-Gly-Gly-His-Lys-NH_2_) is a peptide that includes amino acid sequence GHK (H-Gly-His-Lys-OH), while IM2 (H-Arg-Asp-Lys-Val-Ala-Arg-OH) is a derivative of Imunofan (IM, H-Arg-Asp-Lys-Val-Tyr-Arg-OH). IM is a hexapeptide with both antioxidant and immunomodulatory properties. In prior research, we found that IM enhanced wound healing in a mouse model of dorsal skin damage and stimulated skin cell proliferation in vitro. It did not exhibit any concurrent cytotoxic or allergenic qualities concurrently^11^. Another wound healing peptide is GHK, a copper-binding tripeptide that naturally occurs in plasma and possesses pro-angiogenic properties. It promotes the secretion of trophic factors by mesenchymal stem cells and stimulates collagen and decorin synthesis^12^. Given the broad spectrum of beneficial activities of peptides, recent research has focused on incorporating them into biomaterial-based delivery systems to enhance and prolong their therapeutic effects in wound healing. Peptide gradual-release systems can be developed by combining GHK peptides with hydrogels, such as RADA16-I. Additionally, in animal studies, RADA16-I (Ac-(Arg-Ala-Asp-Ala)_4_-NH_2_) with GHK and KGHK (H-Lys-Gly-His-Lys-OH) sequences was found to promote wound healing^13^.

In this work, we developed a cosmetic formulation comprising the novel peptides NE1 and IM2. In vitro tests of physicochemical properties, microbiological purity, and biological activity of the designed formulation were conducted. In addition, volunteer application tests were implemented. Our analyses demonstrated the safety of the tested formulation and its pro-regenerative and caring properties towards healthy as well as sensitive skin. The other goal was to develop an innovative formulation that is easy to use and allows for painless, even, and controlled application to the affected skin. This was achieved by preparing the formulation in an aerosol form.

## Methods

### Peptide synthesis

IM2 was synthesized with the sequence RDKVAR and the NE1 with the sequence GHKGGGAAPVGGGHK on a solid phase by following o the methodology of Fmoc chemistry and using a P11 synthesizer (Activotec). For synthesis, R RAM (TentaGel®) amide resin was used for NE1 with a loading degree of 0.18 mmol/g and Fmoc-L-Arg(Pbf)-TentaGel S AC for IM2. A mixture of 88% trifluoroacetic acid (TFA) (SigmaAldrich) + 5% H2O + 5% phenol + 2% triisopropylsilane (TIPSI) (SigmaAldrich) (v/v/v/v) was used to detach the peptide from the carrier and simultaneously remove of side-protecting groups. The reaction was performed for 3 h, then the resin was drained, and the filtrate was evaporated. Cold diethyl ether was poured onto the obtained precipitate, which was then diluted, and centrifuged (20 min). Next, the product was dissolved in water and lyophilized. Additionally, chromatographic analysis was performed using reversed-phase high-performance liquid chromatography (RP-HPLC) was performed on a chromatographic column (Luna 5 µm C18, 100 Å, 250 × 4.6 mm (Phenomenex). The conditions were as follows: phase system A: 0.1% TFA in water, B: 0.1% TFA in 80% acetonitrile in water, flow rate 1 ml/min, UV detection at 223 nm.

The trifluoroacetic counter ion was replaced with an acetate ion using Strata C18-E column (55 µm, 70 Å) (Phenomenex). The peptide was applied to an equilibrated column, washed with 0.1 M aqueous ammonium acetate solution and eluted with a 0.1 M solution of ammonium acetate in methanol. The obtained eluate was evaporated using a vacuum evaporator, and the excess ammonium acetate was desublimed on a lyophilizer. To remove the residual ammonium, the lyophilizate was re-dissolved in water and re-lyophilized.

### Peptide stability analysis

The stability analysis of IM2 and NE1 was performed at 1, 2, 3, 6 and 24 h. IM2 and NE1 were incubated in milliQ water at 37 °C and continuous shaking at 300 rpm. The samples collected at the respective time points were analyzed by high-performance liquid chromatography (HPLC) using a Phenomenex Luna C18(2) 4.6 × 250 mm, 5 µm, and 100 Å column with a PDA detector. The systems were 0.1% solution of trifluoroacetic acid (TFA) in water (A) and 0.1% solution of TFA in 80% ACN (B). The analysis was conducted using a linear gradient of 5–100% B in 60 min at a flow rate of 1 mL/min. Calculations were made using the Shimadzu LC-Solution software (Shimadzu).

### Preparation of composition

A formulation was prepared that included IM2 and NE1 at a concentration of 10 µg/ml, 1% poloxamer p-407, and 1–1.5% Euxyl ® K712 food preservative in the form of a mixture of potassium sorbate and sodium benzoate and water.

For in vitro cell culture analysis the composition was prepared in sterile DMEM HG medium and did not contain preservatives. IM2 and NE1 were sterilized under UV lamp in lyophilized form. P407 was sterilized with gamma radiation.

### Peptide release analysis

The transdermal diffusion of IM2 and NE1 was studied using Phoenix Dry Heat Systems. The acceptor and donor chambers were separated by a Strat-M® (Merck) membrane that mimics human skin. A volume of 400 μl of the formulation was applied to the donor chamber, while the acceptor chamber was filled with phosphate buffer and heated to 37 °C with constant mixing at 300 rpm. At 28 predetermined time points over 420 min, 200 μl of solution was withdrawn from the acceptor chamber. The chamber was then refilled with 200 μl of fresh PBS solution. The samples collected were analyzed by HPLC. The quantification of IM2 and NE1 was performed using individual calibration curves for each peptide. Chromatographic separation was achieved on a Kinetex C8 column (2.6 μm, 100 Å, 2.6 × 100 mm, Phenomenex) with a linear gradient of 5–100% solvent B over 15 min at a flow rate of 0.5 mL/min. The mobile phases were as follows: (A) 0.1% TFA)in water, and (B) 0.1% TFA in acetonitrile (ACN, 80%). Detection of peptides was conducted using a photodiode array (PDA) detector, and data were analyzed with LCsolution software (Shimadzu).

### Rotational viscosity test

Poloxamer-407 solutions were prepared at concentrations of 0.5%, 1%, 1.5%, 5%, 10%, and 15% w/v by slowly adding the weighted amount of polymer into cold deionized water (containing previously dissolved peptides) and gently rocking it until it completely dissolved. For the preservative-containing samples, 1.5% w/v preservative (Euxyl ® K712) was added to each concentration of poloxamer solution and mixed until it was homogenous. All the solutions were stored overnight at 4 °C to ensure the complete hydration of the polymer before analysis.

The dynamic viscosity measurements were performed using ViscoQC 300 (Anton Paar, Austria) equipped with a CC18 spindle and a PTD100 temperature unit. The PTD100 temperature unit was installed in the viscometer. An empty measuring cup was used, and different media were poured, which were kept at 4 °C. Each sample was equilibrated at the starting temperature for 5 min before measurement. The viscosity values (in mPa·s) were automatically recorded by the instrument at each temperature point. The viscosity temperature profiles were plotted for each formulation using GraphPad Prism. All the measurements were taken in triplicates, and the mean values were reported.

### Cell isolation

Skin fragments were collected during routine surgical procedures and placed in phosphate buffered saline (PBS) (Sigma Aldrich) with 100 units/ ml penicillin and 100 µg/ml streptomycin solution (P/S) (Sigma Aldrich). The procedure was accepted by the Bioethics Committee for Scientific Research at the Medical University of Gdansk (NKBBN/333/2022). The skin fragments were then cut into smaller pieces and digested with dispase (6 units/ml) (Sigma Aldrich) overnight at 4 °C. The next day, the epidermis was removed and the obtained dermis fragments were placed on 6-well plates in Dulbecco’s Modified Eagle Medium with high glucose 4,5 g/L (DMEM HG) (Sigma Aldrich) supplemented with 10% fetal bovine serum (FBS) (Sigma Aldrich) and 1% P/S (DMEM HG complete medium). The medium was replaced every 2 days. After 2 weeks, when fibroblasts were observed in the wells, the explants were removed.

### Cell culture

The 46BR.1N cell line (Sigma Aldrich), HaCaT cell line (DKFZ, Heidelberg, Germany)^14,15^ and primary dermal fibroblasts were cultured in DMEM HG with 10% FBS and 1% PS umder standard conditions (37 °C, 5% CO_2_). The medium was changed every 2 days. When the cells obtained 80% confluence, they were passaged with trypsin (Sigma Aldrich), centrifuged, and seeded into new culture bottles. Experiments with primary fibroblasts were performed at the fourth passage.

### Proliferation analysis

The 46BR.1N cells, HaCaT cells and primary dermal fibroblasts were seeded in 5000 cells/per well in 200 µl DMEM HG complete medium. For the control and test samples, the medium was replaced with DMEM HG containing 1% PS the following day. The cells were stimulated with compositions devoid of food preservatives at concentrations of 100%, 75%, 50%, and 25%. Additional controls were designed to contain solely IM2 or NE1 peptides, a combination of IM2 and NE1 (IM2 + NE1), and poloxamer p407 alone at concentrations equivalent to those in the 25%, 50%, 75%, and 100% compositions. The cells cultured in a standard DMEM HG medium with 10% FBS served as the positive control. After 24 h of incubation, the XTT (Sigma Aldrich) test was performed according to the manufacturer’s instructions. A volume of 100 µl of cell culture medium was removed, and a mixture of XTT and PMS reagents (50:1 v/v) was added to each well. Spectrophotometric analysis was conducted at 450 nm after 4 h of incubation. The results are presented as % of the control.

### Cytotoxicity analysis on skin fragments

The skin fragments (7 × 7 mm) were placed in 5 ml falcon tubes containing 5 ml of DMEM HG medium (control), p407 or composition. AN additional positive control with DMEM containing 1% TRITON X was performed. The fragments were incubated for 24 h under standard conditions. The skin fragments were them used for histological analysis, and 100 μl of medium from each falcon tube were transferred to a 96-well plate. The LDH (Sigma Aldrich) reagent mixture was then added, and after 45 min, a spectrophotometric reading was taken at 450 nm.

#### Migration analysis

The 46BR.1N, HaCaT cells and primary fibroblasts were seeded in 4-well inserts (Ibidi) at 20,000 cells/well in DMEM HG complete medium. After 24 h, mitomycin C (Sigma Aldrich) was added for 2 h to block cell proliferation. Then, the medium was exchanged to DMEM HG and the cells were stimulated with food-preservative-free composition in 100% concentration. Additional controls containing IM2 + NE1 peptides or p407 alone in concentrations identical to the concentrations in composition were performed. The cells stimulated with DMEM HG with 10% FBS constituted a positive control. After 24 h, the medium was removed, 4% paraformaldehyde (PFA) was added for 30 min for cell fixation and the cells were stained with crystal violet at a concentration of 0.05% for 15 min. The obtained results were analyzed with a Leica DMIL LED inverted microscope and GRAPHAX software.

#### Cytokine analysis

The 46BR.1N cells, HaCaT cells and fibroblasts were seeded in 5000 cells/per well in 200 µl DMEM HG complete medium. The next day, the medium was changed to DMEM HG with 1% PS for the control and test samples, and the cells were stimulated in a combination of IM2 and NE1 peptides, poloxamer p407 and composition without food preservatives at 100% concentration. For FBS control, the medium was replaced by the DMEM HG completed medium. After 24 h, the medium was collected and frozen. On the day of the experiment, the supernatant was unfrozen, and Luminex Multiplex analysis was performed according to the manufacturer’s recommendations (Merck) for IL-6, IL-8, VEGF-A, TNF-alpha, and FGF-2 analytes.

#### Histological analysis

The human skin samples were incubated with p407 and composition at 37 °C for 24 h. After incubation, the samples were fixed in 3.7% formalin. Following a seven-day period of fixation, these samples were rinsed under running water. To dehydrate the fixed tissue fragments, the were subjected to a series of alcohols with progressively higher concentrations. They were then incubated in xylene, followed by incubating them in a paraffin solution at 60 °C and embedding them in paraffin. After the paraffin-embedded tissues solidified, it was possible to cut them into thin sections. (5 μm) using a microtome. The sections with the material were placed on glass slides and left to dry. The prepared slides were stained using Masson’s method with Goldner’s modification and a standard stain used in histology: hematoxylin and eosin. After staining the slide, the material was mounted with DPX solution and a coverslip. The stained slides were analyzed using a light microscope.

#### Physicochemical analysis

The physicochemical tests of the formulation, including preservatives, were conducted in an outsourced laboratory. The tests measured density, viscosity and pH.

#### Microbial purity

The composition with preservatives was subjected to the microbiological purity test and the challenge test at an outsourced laboratory in accordance with PN-EN ISO 11930:2019. In the first stage, the neutralization efficacy of the product’s antimicrobial activity was evaluated for strains of *Streptococcus aureus* (ATCC 6538), *Escherichia coli* (ATCC 8739), *Pseudomonas aeruginosa* (ATCC 9027), *Candida albicans* (ATCC 10231) and *Aspergillus brasiliensis* (ATCC 16,404). Eugon LT 100 neutralizer was used, and the tests were conducted for a sample dilution of 10^–1^. In the second stage, a challenge test was performed. Nephelometrically calibrated suspensions of the microorganisms described above were used to contaminate the test sample. The density of the suspensions ranged between 1 × 10^7^and 1 × 10^8^ CFU/ml for bacteria and between 1 × 10^6^and 1 × 10^7^ CFU/ml for fungi. The results were defined as the value of microbial reduction in the test sample at intervals.

#### Stability and compatibility with the packaging

Conducted in an outsourced laboratory, the stability and packaging compatibility tests evaluated thesample form, pH, presence of mechanical impurities in the composition, stability at − 5 °C, + 18 oC and + 38 o°C, batch number labeling, stability of the preservative system according to ISO 11930:2019, and the compatibility of the mass with the package.

#### Local skin tolerance analysis

An external laboratory conducted a dermatological examination on the prepared composition to assess the topical dermal tolerance of the cosmetic product in healthy volunteers and to identify any sensitizing or irritating properties of the preparation. After conducting a physical examination, a specialist dermatologist selected participants based on inclusion and exclusion criteria, which were in accordance with the 1964 Declaration of Helsinki (with subsequent modifications), Polish and EU regulations, and COLIPA guidelines. Twenty healthy volunteers, both men and women, with ages ranging from 18 to 77 years, were recruited. They all had dry skin with a propensity for atopy, allergies, and irritation. Two of them had undergone radiation and chemotherapy, two others had psoriasis, and four had a history of diabetes. During the test, they were advised to refrain from consuming antihistamines or other pharmacological agents (both systemic and topical) that could potentially impact the results, and from undergoing skin irradiation. No lesions were present on the skin at the patch test application site. After they were informed of the test procedure and given a briefing, the participants gave their consent to participate in the study. Patch (epidermal) tests were then conducted. The composition was applied in commercial form to blotting paper discs, which were fixed with a hypoallergenic patch on the skin of the back in the interscapular region. The samples were removed after 48 h. The first reading was taken after 24 h, 48 h, and 72 h after the application of the composition. The evaluation was conducted in accordance with the prevalent dermatological testing scale, and a dermatologist assessed the participants’ subjective feelings and skin before, during, and after the test.

#### Application analysis

An external laboratory conducted dermatological testing of the prepared composition. The participant selection was done according to the principles described in the previous section. All the participants gave their informed consent to participate in the study. They were provided with one package of the composition and were required to do the following: observe and document the functional properties of the tested preparation in detail; abstain from using any other products with an identical or analogous purpose for four weeks; immediately discontinue the use of the preparation in the event of any negative symptoms at the site of use and report them to the investigator; report for follow-up examinations during and immediately after the study. The participants implemented the preparation in accordance with the manufacturer’s general guidelines. Findings were obtained by conduction direct interviews with the participants during the application process.

## Results

### Peptide stability

The stability of IM2 and NE1 was evaluated by monitoring the percentage of peptides remaining over a 24-h incubation period.

**Fig. 1:**
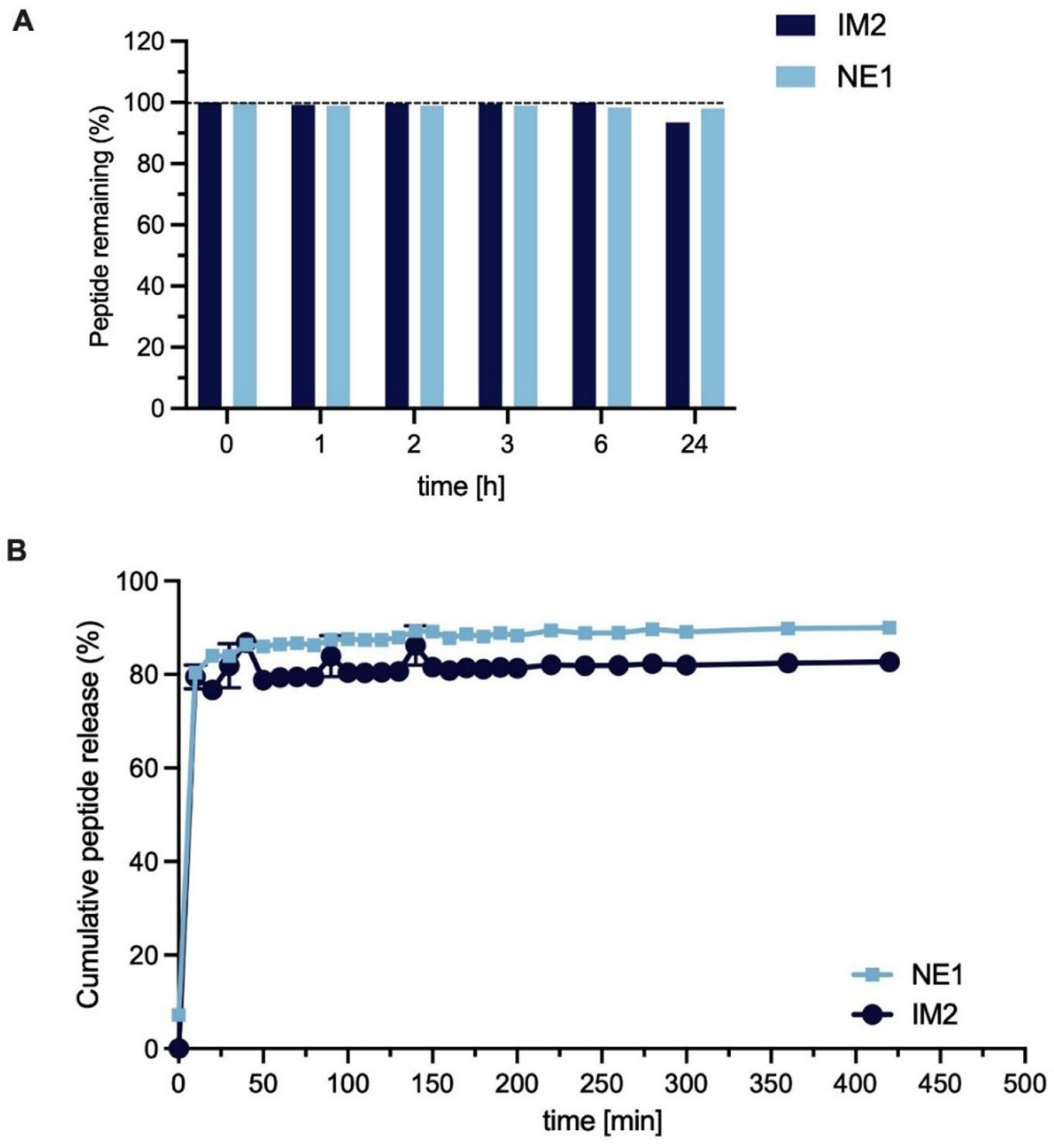
Physicochemical analysis of peptides. (**A**) Stability of peptides IM2 and NE1 expressed as the percentage of peptide remaining over time. (**B**) The cumulative release profiles of peptides IM2 and NE1 from a 1% poloxamer-407 formulation over 420 min.

Both peptides showed excellent stability, maintaining values close to 100% during the first 6 h (Fig. 1A). After 24 h, only a slight decrease was observed, which was more pronounced for IM2 (approximately 93%) compared to NE1 (98%), which remained closer to the initial level. These results indicate that IM2 and NE1 retain their integrity under the tested conditions for at least 24 h.

### Peptide release analysis

The release profiles of IM2 and NE1 from a 1% p407 formulation were evaluated over a period of 420 min and expressed as cumulative peptide release (%).

Both peptides exhibited a pronounced initial burst release within the first few hours, reaching approximately 75%–80% of the total peptide load (Fig. 1B). Following this rapid release phase, the profiles entered a plateau stage, indicating that further diffusion of the peptides from the p407 matrix was limited. Over the extended incubation period, NE1 displayed a slightly higher cumulative release, stabilizing at around 87–90%, whereas IM2 reached around 82%–83%. This difference suggests that NE1 is more readily released from the p407 network compared to IM2, potentially reflecting the differences in their physicochemical properties or interactions with the carrier. The slight decreases observed in the IM2 curve at selected time points (e.g., 20, 40, 90 and 140 min) arise from experimental variability, including sampling and analytical measurement.

### Dynamic viscosity properties

**Fig. 2:**
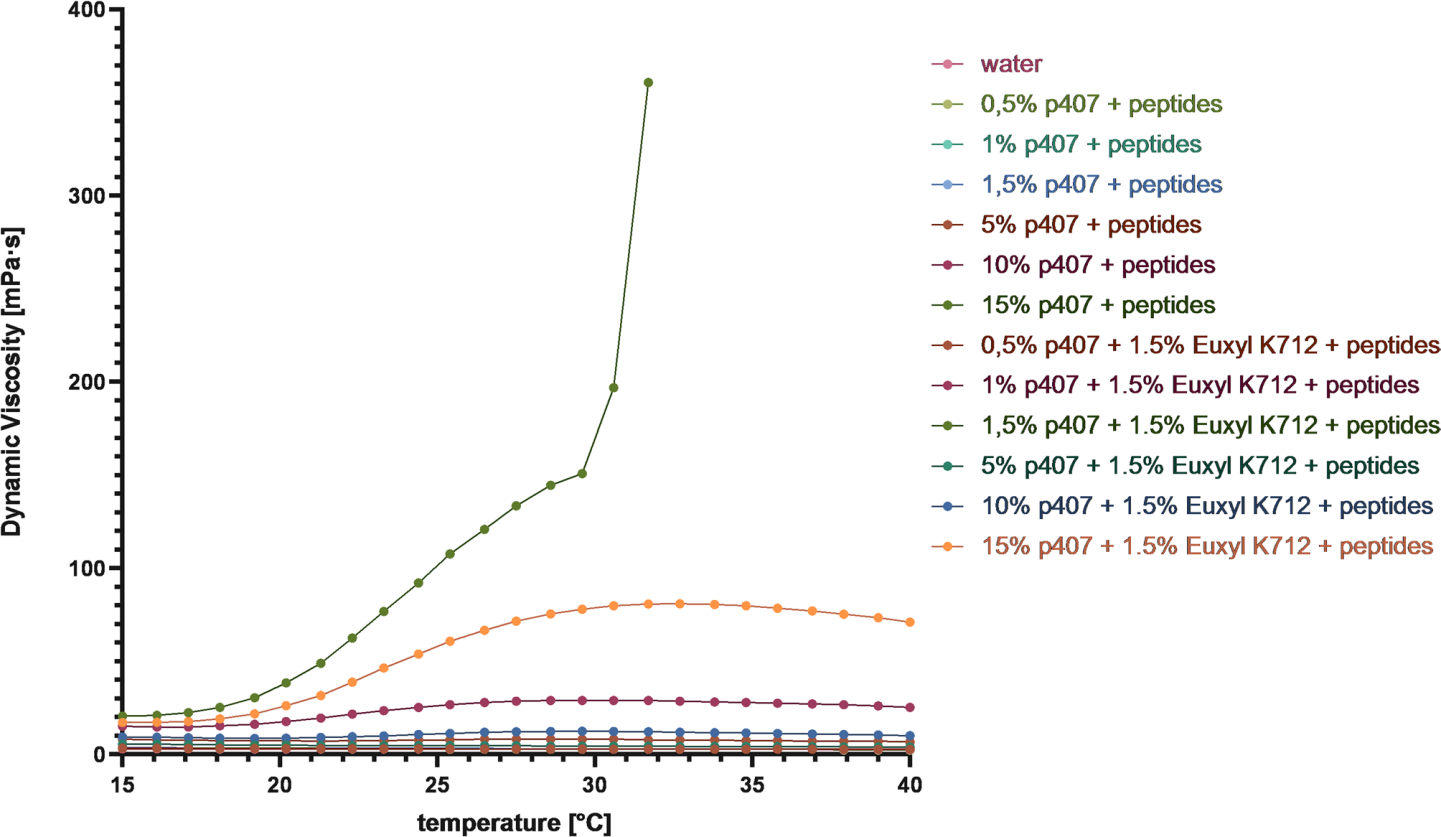
The dynamic viscosity of aqueous poloxamer-407 solutions with and without preservatives as a function of temperature (15–40 °C). All the solutions contained peptides except the control (water).

The dynamic viscosity profile of the different concentrations of poloxamer-407 with peptides and preservatives revealed changes in dynamic viscosity after the addition of preservatives (Fig. 2).

Water’s viscosity remained nearly constant across the temperature range (~ 1–2 mPa·s), serving as the control. In low concentrations of p-407 (0.5–1.5%) no clear thermoresponsive gelation was observed. At higher concentrations (5–10%) viscosity was higher than that of water. At 10%, there was a more pronounced rise, but the slope remained relatively smooth, suggesting micellization and early structural organization but not strong gelation. The addition of preservative slightly lowered the viscosity of these formulations. In high concentrations, the viscosity of p407 rose sharply, starting at 20–25 °C and increasing dramatically above 30 °C, exceeding 350 mPa·s at ~ 32 °C. This reflects the thermogelling transition of p407: micelles aggregate and form a network as the temperature rises with the preservative. The viscosity also increases, but the curve is flatter and peaks around 70 mPa·s before declining slightly, suggesting that the preservative interferes with network formation and reduces the strength of the gel.

### Cell proliferation analysis

In the European Union, animal testing is prohibited, and it raises countless ethical concerns worldwide. In vitro methods are thus essential for the early testing of the biosafety of newly developed cosmetic formulations before they are forwarded into skin tolerance and application tests. In this study, we performed an in vitro analysis of the effects of the designed composition and its components on the proliferation of human skin cells.

**Fig. 3:**
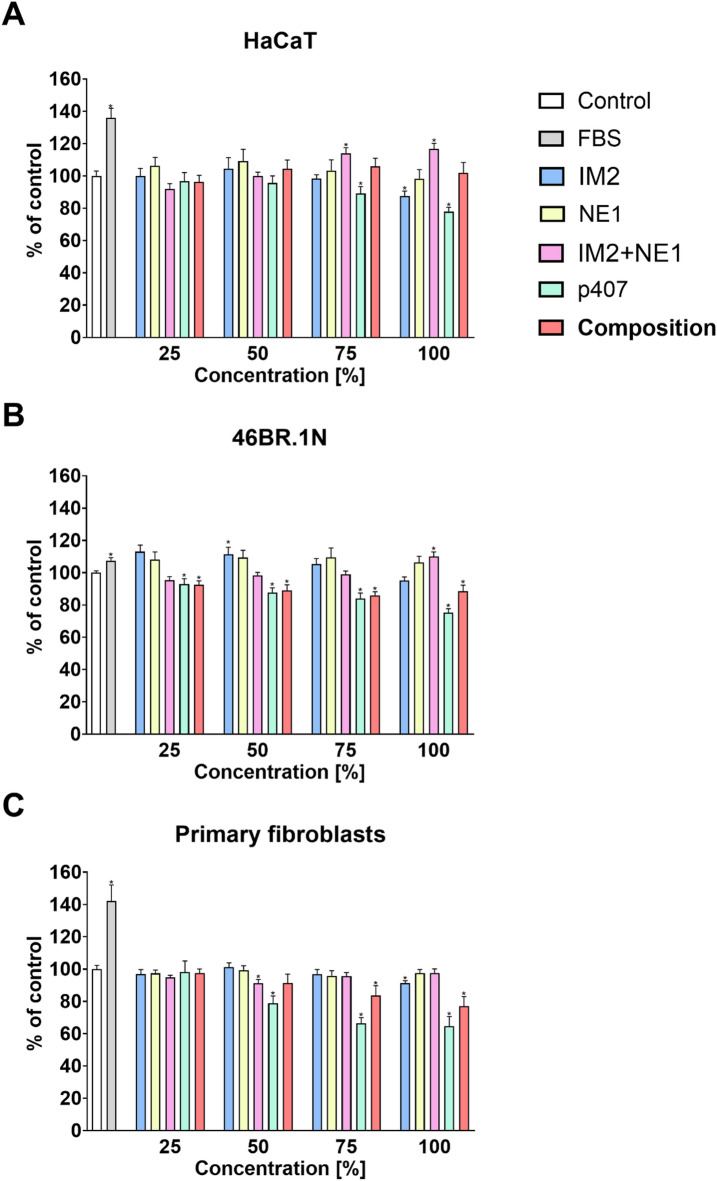
The effect of composition and its components on the proliferation of human cells: HaCaT keratinocytes (**A**), 46BR.1N fibroblasts (**B**) and primary dermal fibroblasts (**C**). The graphs present the mean ± SEM from three independent experiments performed in quadruplicate. *indicates statistically significant differences, Mann Whitney U test, p < 0.05). FBS was used as the positive control.

The proliferation of the HaCaT cell line (Fig. 3) was observed to increase by approximately 15% following stimulation with IM2 + NE1 peptides at the levels comparable to those in the 100% and 75% compositions in our study. Approximately 10% and 20% decrease in concentrations equivalent to those in 75% and 100% composition were observed for p407, which is employed as an emulsifying agent in cosmetic and industrial products^16^. However, we did not observe any inhibition of proliferation after keratinocytes were stimulated using the designed composition. After stimulation with IM2 peptide at concentrations equivalent to those in the 25% composition, 46BR.1N fibroblasts exhibited a mild but statistically significant increase in their proliferation (approximately 10%). A comparable effect was visible for the stimulation with IM2 + NE1 peptides in concentrations that were the same as in the 100% composition. The viability of 46BR.1N was slightly reduced (5–25%) in all concentrations tested for p407. The designed composition also slightly (5–15%) reduced 46BR.1N viability in all tested concentrations. P407 was found exert the most potent inhibitory effect on primary dermal fibroblasts, with concentrations equivalent to those in the 25%, 50%, and 75% compositions ranging from 20 to 35%. The inhibitory effect was, however, less potent in the designed composition, as evidenced by a 25% decrease in fibroblast viability. Cytotoxic effect is defined as the reduction in viability of over 30%, as per ISO 10933–5:2009. Our results thus confirmed the safety of the peptides and the composition for skin cells.

### Cytotoxicity analysis and skin histology

Biosafety analysis was continued with the use of the LDH cytotoxicity method and histological staining. The skin samples obtained from five individuals were incubated with the designed composition and p407 alone in a concentration equal to the 100% composition.

**Fig. 4:**
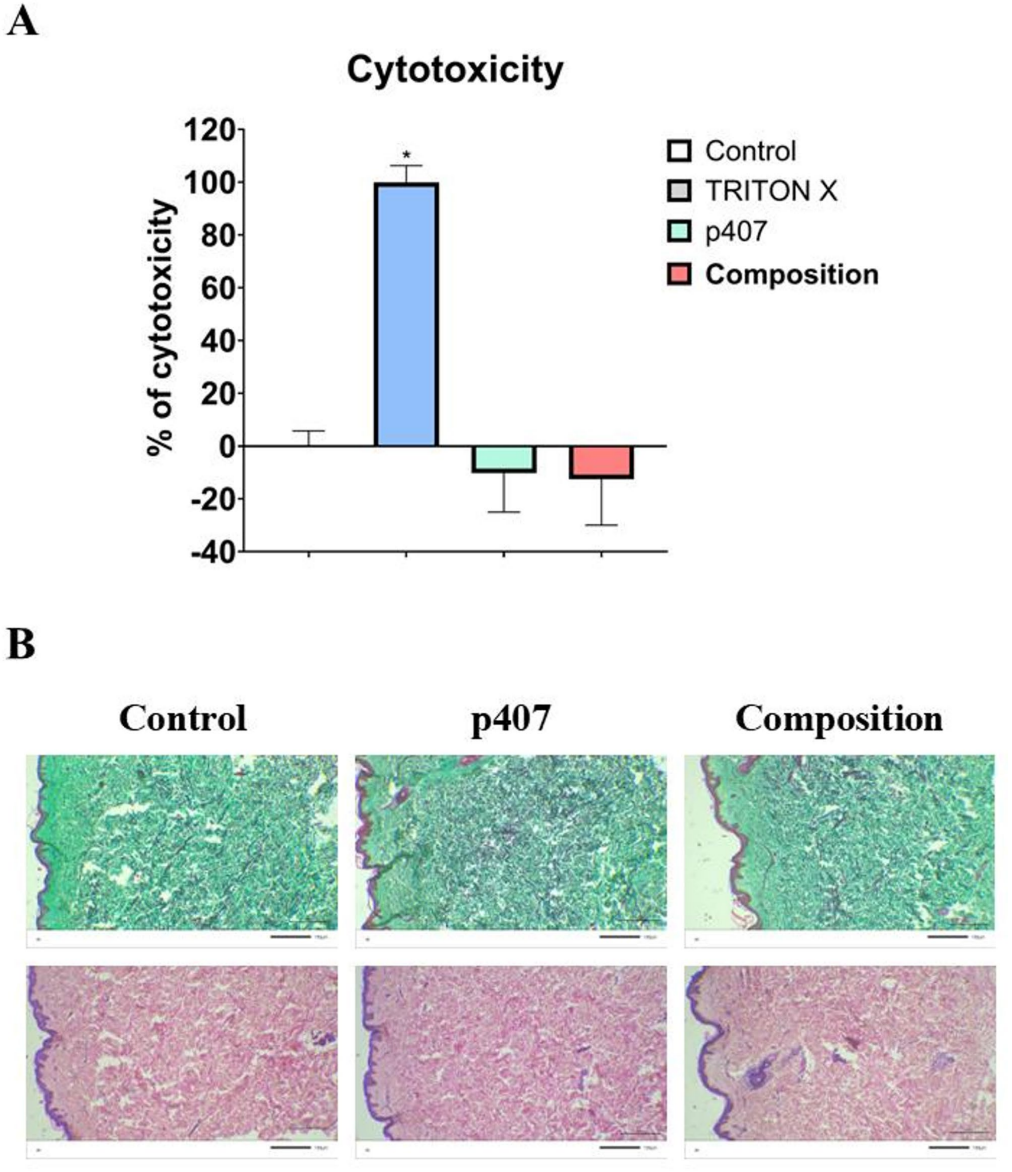
Biosafety confirmation. (**A**) Cytotoxicity of p407 and the composition on skin explants. The graphs present the mean ± SEM from five independent experiments performed in quadruplicate. *indicates the statistically significant differences (Mann Whitney U test, p < 0.05). TRITON X was used as the positive control for maximum LDH release. (**B**) The histological staining of the skin explants incubated with p407 and composition. The upper panel shows Masson’s trichrome staining, and the lower panel shows hematoxylin—eosin staining.

The histological analysis of the skin samples incubated with p407 and the tested composition did not reveal any differences between the control and treated skin (Fig. 4).

The obtained results (Fig. 4) also confirm the safety of the designed composition. Overall, the mean cytotoxicity was less than 0. However, it should be added that we observed inter-individual differences; in three samples result was significantly below 0, while cytotoxicity was observed in the remaining two samples.

### Migration analysis

Cell migration is a key process during wound healing and the regenerative process that allows for the rapid restoration of the skin physical barrier^17^.

**Fig. 5:**
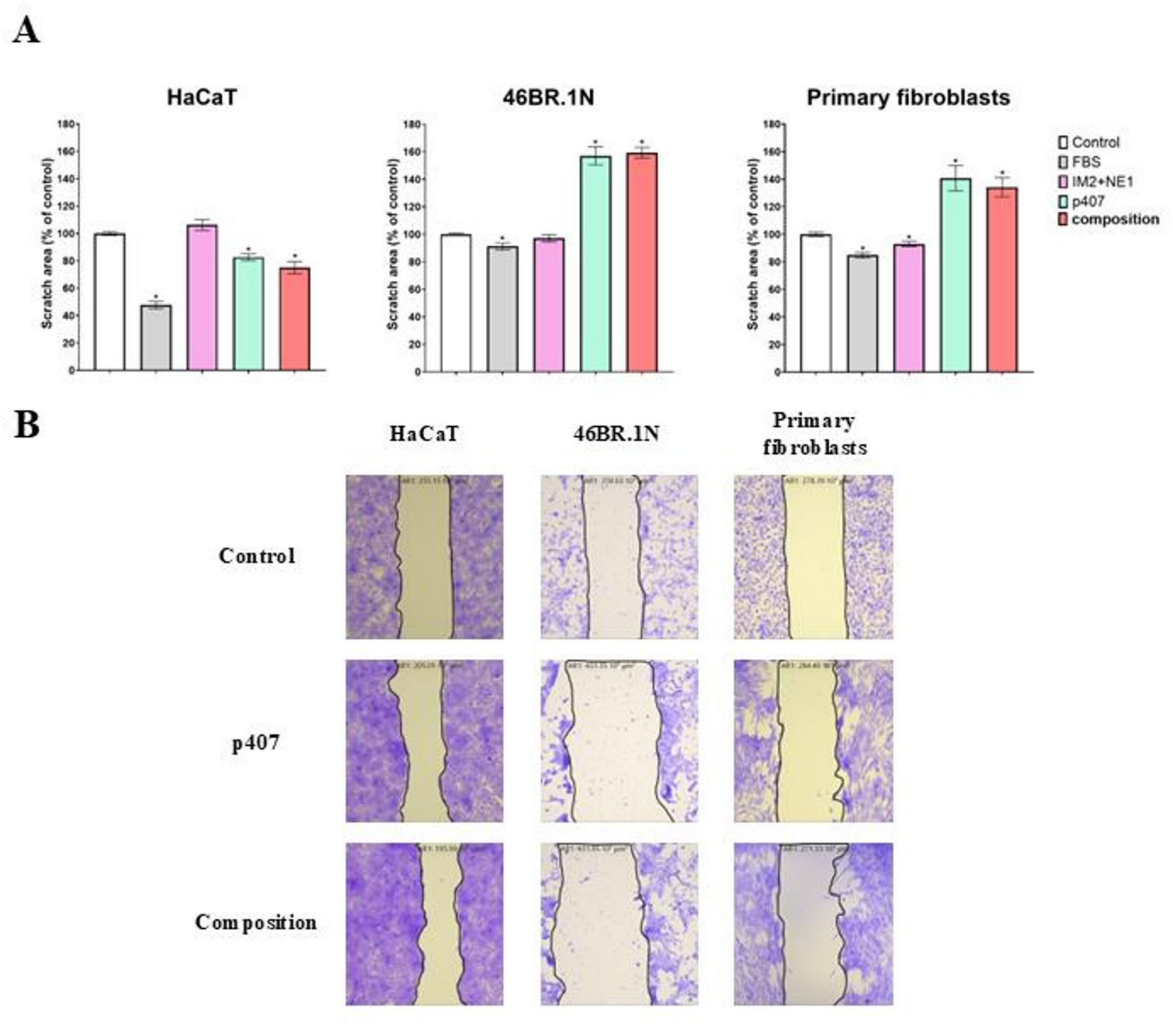
Cell migration analysis. (**A**) The effect of composition and its components on the migration of human cells: HaCaT keratinocytes (left chart), 46BR.1N fibroblasts (middle chart) and primary dermal fibroblasts (right chart). The graphs present the mean ± SEM from three independent experiments performed in quadruplicate. * indicates statistically significant differences (Mann Whitney U test, p < 0.05). FBS was used as the positive control; (**B**) Representative images of cell migration analysis.

For HaCaT keratinocytes (Fig. 5), a statistically significant stimulation of migration was observed for p407 (about 18%) and the prepared cosmetic product (about a 25% reduction in scratch area). The combination of the IM2 + NE1 peptides revealed no effect on cell migration. Interestingly, the inhibition of migration was observed in fibroblasts.

### Evaluation of cytokine levels

During regenerative processes, skin cells secrete various cytokines and growth factors. They participate in the regeneration process, regulate cellular responses, migration, and proliferation and enable the maintenance of the skin’s function and structure^18,19^.

**Fig. 6:**
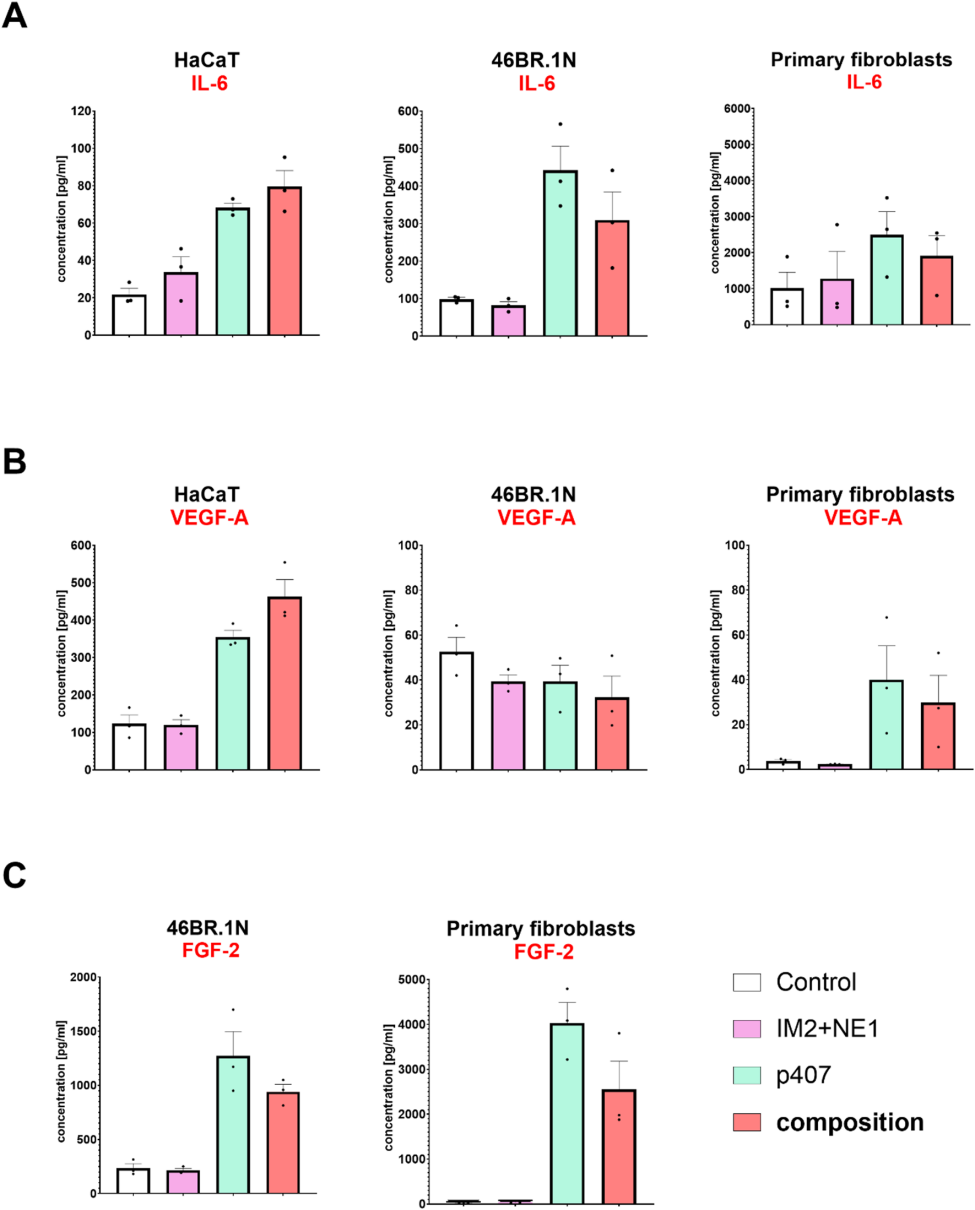
Levels of IL-6 (**A**), VEGF-A (**B**), and FGF-2 (**C**) after IM2 + NE1, p407 and composition stimulation of the skin cells evaluated with Luminex technology. The graphs present data from three independent experiments. No statistically significant differences were detected (Mann Whitney U-test, p < 0.05).

**Fig. 7:**
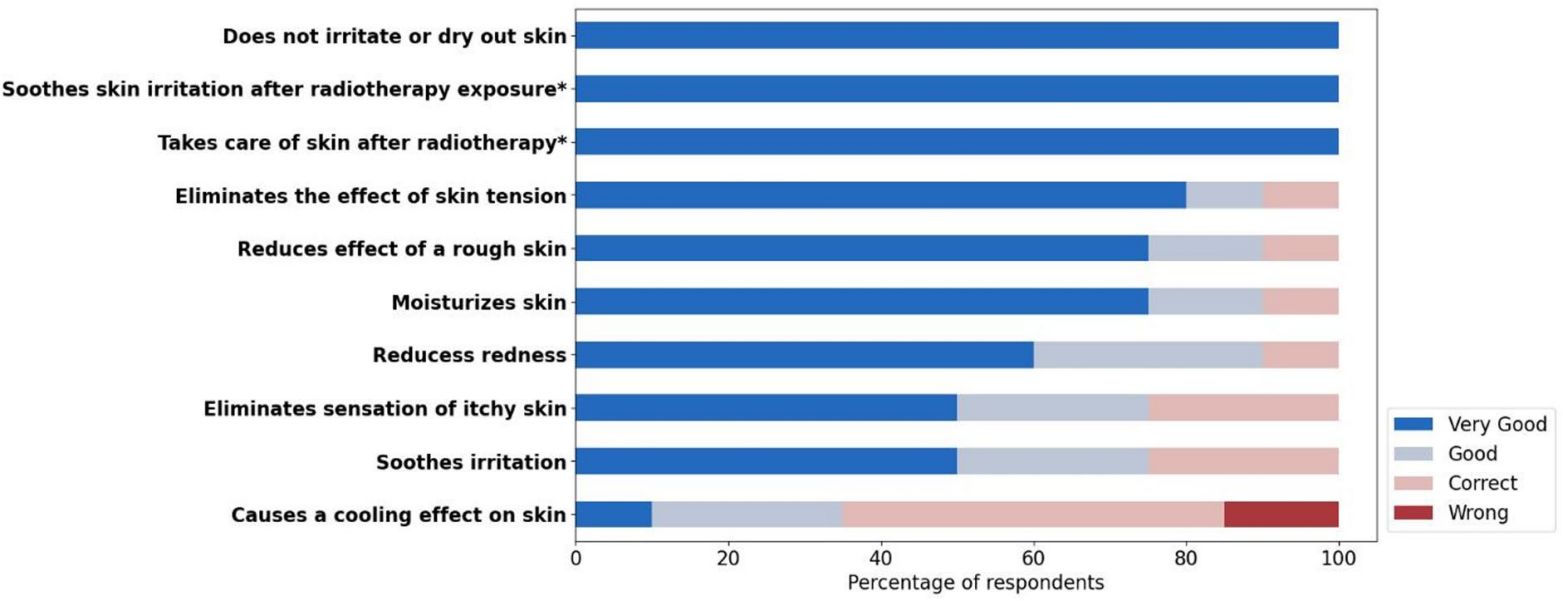
Application evaluation of composition. The graph show the percentage of respondents with a given score.*- two participants had undergone radiotherapy.

Despite no statistically significant differences in the Luminex analysis, there were some changes. Upon stimulation with p407 and composition, HaCaT keratinocytes exhibited an increase in IL-6, VEGF-A (Fig. 6), eotaxin, IL-8, and IL-1a concentrations (Fig. 1S). The stimulation of 46BR.1N with p407 and composition led to an increase in the levels of IL-6, FGF-2, and eotaxin, as well as a decrease in the levels of G-CSF (Fig. 1S). For primary dermal fibroblasts, stimulation with p407 and composition resulted in an elevated secretion of IL-6, VEGF-A, FGF-2 (Fig. 7), and eotaxin as well as a decrease in TNF-alpha and G-CSF secretion (Fig. 1S). The secretion of IL-6, VEGF-A, and FGF-2 was increased in primary dermal fibroblasts by stimulation with p407 and composition (Fig. 6), while TNF-alpha and G-CSF secretion decreased (Fig. 1S).

### Microbiological purity, physicochemical and stability tests

For a cosmetic product to be commercialize, biological purity, stability, and packing compatibility analyses are required. The neutralization efficiency of the preservative system in the test sample was determined for the neutralizer: Eugon LT 100 at a sample dilution of 10^–1^. The assumption of Nvf ≥ 0.5 Nvn was met for all the test strains used in the neutralization study. The efficiency of the neutralizer was assessed at over 50%. (Table 1S). In the challenge test, in accordance with the assumptions of the PN-EN ISO 11930:2019 standard, nephelometric-calibrated suspensions of test microorganisms (N) with a density in the range of 1 × 10^7^–1 × 10^8^ unit/ml for bacteria and 1 × 10^6^–1 × 10^7^ unit/ml for fungi were used to contaminate the test samples. The results presented in Table 2S meet these assumptions. The analysis demonstrated that the prepared formulation meets the necessary criteria A and B of antimicrobial protection effectiveness for the test strains utilized in the study, as outlined in the PN-EN ISO 11,930:2019 standard. The results (Tables 3S, and 4S) confirm the preservative properties of the product.

In addition, the tests on the stability of the product at three temperature points (− 5 °C, + 18 °C, + 38 °C) showed no signs of mass destabilization, reactivity with packaging, or other changes. Based on the tests, the product was determined to be stable and to have preservative properties (Table 6S). The minimum durability date was marked as 24 months from the date of production.

Furthermore, the viscosity of the product was assessed as 4.43 ± 0.042 [mPas]. The density for the product was 0.9888 [g/ml], and the pH was 5.5.

### Local skin tolerance and application analysis of the developed product

The composition was prepared in the form of an aerosol. The liquid form of the composition is advantageous, as it enables its atomization, which enables painless and uniform application of the substance to sensitive skin following radiotherapy, burns, and the action of other irritating factors (such as temperature and sun radiation).

As per the dermatological test, the composition did not exhibit any irritant or sensitizing properties. In the group of 20 individuals tested (all with a history of allergy), no positive skin reactions were observed.

After the application tests, the composition was noted to be highly tolerated (Table 1). Furthermore, it was found to effectively moisturize and take care of sensitive skin, alleviate redness and the effects of rough skin, eliminate the sensation of itching and the effects of skin tension, and soothe irritation (Fig. 7). Additionally, evaluated product was found to satisfy the requirements of functionality. To a very good degree, 50% of the respondents rated the composition as soothing irritation. Redness was reduced to a very good level in 60% of the participants. The composition was reported to be effective in alleviating skin irritation and caring for the skin after radiotherapy by two of the 20 patients who participated in the tests and had previously experienced skin damage. Moreover, 75% of the participants reported that the composition moisturized the skin at a very high level, and all the participants reported that it did not irritate or dry out the skin. Moreover, 80% of the participants indicated that the effect of skin tension was eliminated, while 75% reported that the effect of rough skin was significantly reduced. Furthermore, all participants reported that the sensation of itchy skin was eliminated, with 50% reporting it as very good and 25% reporting it as good at the correct level.

**Table 1:** Results of the application tests of the composition.

Probant	Age	Sex	Skin type	Results
1	54	M	M	(-) A
2	49	F	S	(-) A
3	18	F	T	(-) A
4	23	F	S	(-) A
5	76	M	M	(-) A, D
6	76	F	M	(-) A, D
7	73	F	N	(-) A, D
8	70	F	S	(-) A, D
9	49	F	S	(-) A, P
10	72	F	M	(-) A, P
11	49	F	S	(-) A
12	49	F	N	(-) A
13	54	M	N	(-) A
14	50	F	M	(-) A, R
15	72	F	S	(-) A, R
16	18	F	S	(-) A
17	18	F	S	(-) A
18	18	F	N	(-) A
19	19	F	S	(-) A
20	23	F	S	(-) A

A—history of allergy, P—history of psoriasis, D—history of diabetes, R-radiotherapy.

Sex: F—female, M—male.

Skin type: N—normal, D—dry, M—mixed, T—oily.

Patch test results assessed by a dermatologist:

(−)—negative result; no reaction.

## Discussion

The largest organ in the body, the skin serves as a protective barrier against pathogens and physical factors, such as UV radiation. Its epidermis, which is its outermost layer, is subjected to a variety of physical factors, including the sun and injuries, which can cause allergies and damage^20,21^. In the modern era, atopic dermatitis, psoriasis, acne, alopecia, bacterial or fungal infections, and scabies are becoming more prevalent^22^. Additionally, the number of cancer patients is increasing at an unprecedented rate, with radiation therapy remaining the primary treatment for 50% of them. However, many patients continue to experience skin issues due to radiation therapy, despite the use of increasingly precise equipment that restricts the exposure of radiation to neighboring tissues^23^. Moreover, consumer interest in new products that are developed using scientific knowledge is consistently increasing, and the cosmetics market is expanding at a rapid pace^24^.

In this study, we introduced a novel cosmetic formulation composed of preservatives, bioactive peptides, and p407 specifically designed for skin care. To evaluate the biological activity and safety of a product developed, in vitro testing was conducted, according to the ISO 10,933–5:2009 guidelines. Additional examinations included dermatological tests with volunteers and physicochemical tests of the composition.

Two peptides were chosen for the product’s composition. NE1 that contains the GHK sequence, which is known for its pro-regenerative and anti-aging properties^25^. IM2 is a derivative of the IM peptide, which showed pro-regenerative activity in our previous research^11^. The cumulative release of the IM2 and NE1 peptides from a 1% poloxamer formulation was assessed over 420 h. Both peptides displayed a biphasic release profile, with an initial burst phase followed by a plateau. Within the first hours, approximately 75%–80% of each peptide was released, ensuring a rapid onset of action. Over the prolonged incubation period, the release stabilized, reaching ≈82%–83% for IM2 and ≈87%–90% for NE1. When considered together in a dual-peptide formulation, these profiles suggest a complementary mode of action. The rapid co-release of both peptides during the burst phase could provide an immediate effect, beneficial for attenuating symptoms of skin damage such as inflammation or epidermal barrier disruption.

The thermoresponsive behavior of p407 was found to be strongly concentration-dependent, with solutions below 15% w/v failing to demonstrate pronounced gelation within the physiological range (20–37 °C). The incorporation of preservatives was found to exert a marked influence at higher concentrations; in particular, a 15% w/v p407 solution exhibited a substantial reduction in viscosity in the presence of preservatives. This observation indicates that certain additives may disrupt the self-assembly process of poloxamer micelles, thereby attenuating gel formation. Based on the viscosity profiles obtained, formulations in the range of approximately 20–100 mPa·s (observed for systems containing 5–10% poloxamer-407) may be considered optimal for topical applications. However, the selection of the final formulation was guided by two principal factors: rheological performance and cytotoxicity, the latter attributable to both poloxamer and preservative. Following topical application, the formulation was required to maintain sufficient fluidity to permit administration via spraying, while simultaneously achieving adequate viscosity to prevent runoff from the skin surface. Subsequent water evaporation during application, combined with the skin’s elevated temperature, increased local polymer concentration and promoted gelation, resulting in the formation of an occlusion film beneficial for the managing dry skin.

The physicochemical analyses confirmed the preservative properties and stability of the designed composition. The safety of the prepared product was verified using XTT analysis of its activity. In all the examined cells, the proliferation inhibition was less than 30%. Intriguingly, the proliferation inhibition in all these cells was greater than that of the composition itself when p407 was used at a concentration equivalent to that of the 100% composition. Poloxamer is a well-known ingredient in cosmetic and medical formulations^16,26^. However, in the literature we can find information about its inhibitory effects in vitro. Hsieh et al.^27^ showed that in Alamar blue assay after 3 h of incubation 23% p407 reduced the viability of human Hs68 fibroblast by about 15%, while 25% reduced the viability by about 20%. On the other hand, 3 h incubation of 20% p407 with EpiDerm skin equivalent did not result in viability reduction. Moreover, the LDH assay did not reveal any cytotoxic effect of 20% p407^28^. In our study, LDH analysis also confirmed the safety of p407 and the prepared cosmetic composition on skin explants. Furthermore, the composition’s safety was further substantiated by the absence of any differences between the control and incubated skin samples in the histological analysis.

Analysis of cell migration indicated that both p407 and the designed composition stimulated the migration of HaCaT keratinocytes in vitro. In normal skin, keratinocytes are the main components of the epidermis and are fundamental to its protective barrier function^29^. Upon injury, keratinocytes are activated, and migration and proliferation are initiated to restore the epidermis’ barrier function ^30,31^. This is why the promigratory activity of the cosmetic composition that has been developed may be advantageous for the protection and regeneration of damaged skin, such as in the case of post-sun or post-radiotherapy exposure or for individuals with epidermal diseases such as atopic dermatitis. However, the inhibition of fibroblast migration was also noted. Fibroblasts are dermal components responsible for the production of ECM elements, such as collagen and elastic fibers, as well as the remodeling of the ECM. This process is integral to the regulation of tissue repair and homeostasis^32,33^. The results indicate that fibroblasts as cells of different origin can respond differently to stimuli. This may be due to their different functions, expression of receptors and activation of cell signaling. Keratinocytes are mainly involved in the process of epithelialization, while fibroblasts in the production of ECM and tissue remodeling. Taking into account their biology, keratinocytes may be more responsive e.g. for a compound that promotes cytoskeletal remodeling, while fibroblast at the same time may show reduced migration due to altered adhesion or matrix related signaling. Therefore, the observed promotion of HaCaT migration and inhibition of fibroblast migration by P407 and the formulation is likely related to cell-type-specific signaling and their functional differences in skin repair processes^34^. Nevertheless, the designed composition is intended for application to the skin’s outer layer.

Luminex analysis of the secretory profile of skin cells after stimulation with designed composition and p407 revealed some differences. An increase in IL-6 levels was visible for fibroblasts and keratinocytes after stimulation with both, p407 and the designed product. IL-6 is released early in response to injury, and its signaling is responsible for the switch to a reparative environment^35^. Although IL-6 has been classified as pro-inflammatory, its function is pleiotropic, and it can express both pro- and anti-inflammatory actions. IL-6 links adaptive and innate immunity, which regulates antimicrobial defense and is known for its protective role in many infections^36,37^. IL-6 also participates in the regeneration of the stratum corneum and skin barrier function, and in maintaining skin homeostasis^38^. Its protective effect against infections and the activation of regeneration may be beneficial in the reconstruction of damaged epidermis. Additionally, a decrease in the secretion of TNF-alpha was observed in primary fibroblasts. The levels of TNF-alpha are reported to be increased in people predisposed to impaired wound healing^39^. Additionally, the elevated levels of TNF-alpha in psoriasis lesions trigger an inflammatory cascade that leads to tissue damage. TNF-alpha can also enhance the production of intercellular adhesion molecule-1 (ICAM1) by keratinocytes and endothelial cells, which may then lead to an increased likelihood of T cell binding and the infiltration of T lymphocytes into the skin. What is more, TNF-alpha is reported to be involved in the skin-aging process, and chronic inflammation, which it mediates, is believed to be responsible for the formation of wrinkles and other signs such as poor hydration or the disintegration of dermis and epidermis junctions^40^. Consequently, the observed effect of TNF-alpha reduction may be advantageous for the maintenance of skin homeostasis. In addition, the study revealed an increase in the secretion of VEGF-A for HaCaT cells and primary fibroblasts, as well as FGF-2 for both types of fibroblasts. This is interesting, because both growth factors are important for skin repair. FGF-2 activates vascular endothelial cells and fibroblasts and promotes wound closure. It has also been reported to have the ability to expedite epithelialization^41^. VEGF is the most important pro-angiogenic growth factor in the skin, but other cells (such as keratinocytes) express VEGF receptors (VEGFR) and are able to respond to the activity of VEGF. For example, it has been found that VEGF can regulate the repair of the epidermal barrier and dermis during the proliferative phase of wound healing^42^. Nevertheless, it is crucial to underscore that no statistical significance was observed, necessitating additional investigation of the identifiable effects in the future. It is worth emphasizing, however, that in further application studies, all the participants confirmed that the applied product did not irritate the skin and exhibit any irritating or sensitizing properties It is also important to consider that in vitro studies expose cells directly to the stimulator, which may increase their sensitivity to the effect. When the product is applied to the skin, cells are enclosed by their niche, which comprises other types of cells, such as immune system cells, and other components of the extracellular matrix. Consequently, their susceptibility to the compositions tested may be slightly different. It is thus imperative to underscore that in vitro studies, while offering valuable insights into the safety and efficacy of novel formulations, cannot replace in vivo studies.

Most importantly, the application test verified that the product is extremely safe. The product was well tolerated, did not exhibit any sensitizing or irritating properties and was deemed useful in taking care of sensitive skin.

The in vitro analyses performed in the study utilized only healthy cell, and future research involving damaged skin models or inflammation-induced model would be beneficial for the development of the product and e.g. its registration as a medical product not a cosmetic one. The future research may also involve larger groups of people with specific skin conditions, e.g. people with skin allergies, psoriasis, diabetes or after radiotherapy, which may show the exact potential of the designed composition in taking care of skin from those groups of patients.

## Conclusion

This research delineated the development of a novel hydrogel cosmetic composition that contains bioactive peptides for potential use on sensitive skin. It was found that the incorporation of peptides into a hydrogel could be a promising approach for the development of novel cosmetics. The aerosol formulation of the product was intended to facilitate its painless and uniform application to irritated or damaged skin and is advantageous over other product present in the cosmetic market. The composition was found to be safe through in vitro analyses, and the physicochemical test confirmed its preservative properties and stability. Moreover, the product’s high tolerance, as evidenced by the absence of irritating or sensitizing properties, was confirmed by the application analyses conducted on the participants. Additionally, its usefulness in skin care was revealed. Since the participants included individuals with sensitive skin, a history of allergies, psoriasis, and even radiotherapy, the product’s high tolerance is very encouraging. The proposed formulation has the potential to be utilized for the daily skin maintenance of individuals with sensitive, allergic, or irritated skin, such as those who have been exposed to the sun. The product shows preliminary potential, which requires confirmation through larger-scale clinical studies. Additional research is required to confirm its efficacy in a broader population of patients with skin damage caused by radiation therapy.

The in vitro analyses performed in the study utilized only healthy cell, and future research involving damaged skin models or inflammation-induced model would be beneficial for the development of the product and e.g. its registration as a medical product not a cosmetic one. The future research may also involve larger groups of people with specific skin conditions, e.g. people with skin allergies, psoriasis, diabetes or after radiotherapy, which may show the exact potential of the designed composition in taking care of skin from those groups of patients.

## Supplementary Information

Below is the link to the electronic supplementary material.

Supplementary Material 1

## Data Availability

Data will be available from the corresponding Author on request.
