# Supplementary material for: Peptide-enriched hydrogel formulation for sensitive and damaged skin: from design to application testing
Source: Sci Rep. 2026 Apr 24;16:19053. doi: 10.1038/s41598-026-49562-4 (PMC13279771; doi:10.1038/s41598-026-49562-4)
Supplement: Supplementary file 1 — Supplementary Material 1 [file 41598_2026_49562_MOESM1_ESM.docx]

Deptuła Milena^1,*^, Zawrzykraj Małgorzata^2^, Sawicka Justyna^3^, Dzierżyńska Maria^3^, Skoniecka Aneta^4^, Czerwiec Katarzyna^2^, Kondej Karolina^5^, Zieliński Jacek^6^, Janus Łukasz^7^, Rodziewicz-Motowidło Sylwia^3^, Pikuła Michał^1,8^

**Peptide-enriched hydrogel formulation for sensitive and damaged skin: from design to application testing**

1. Laboratory of Tissue Engineering and Regenerative Medicine, Division of Embryology, Medical University of Gdańsk, Poland
2. Division of Clinical Anatomy, Medical University of Gdańsk, Poland
3. Department of Biomedical Chemistry, Faculty of Chemistry, University of Gdańsk, Poland
4. Division of Embryology, Medical University of Gdańsk, Poland
5. Department of Plastic Surgery, Medical University of Gdańsk, Poland
6. Department of Surgical Oncology, Transplant Surgery and General Surgery, Medical University of Gdańsk, Poland
7. BioGel Sp. z o.o., Szybowcowa 8a, 80-298 Gdańsk, Poland
8. Department of Biochemistry, Gdańsk University of Physical Education and Sport, Gdańsk, Poland

*- Correspondence: Milena Deptuła, PhD Eng., email: [milenadeptula@gumed.edu.pl](mailto:milenadeptula@gumed.edu.pl), Laboratory of Tissue Engineering and Regenerative Medicine, Division of Embryology, Medical University of Gdansk, Debinki 1, 80-211 Gdansk, Poland; ORCID: 0000-0003-4875-770X


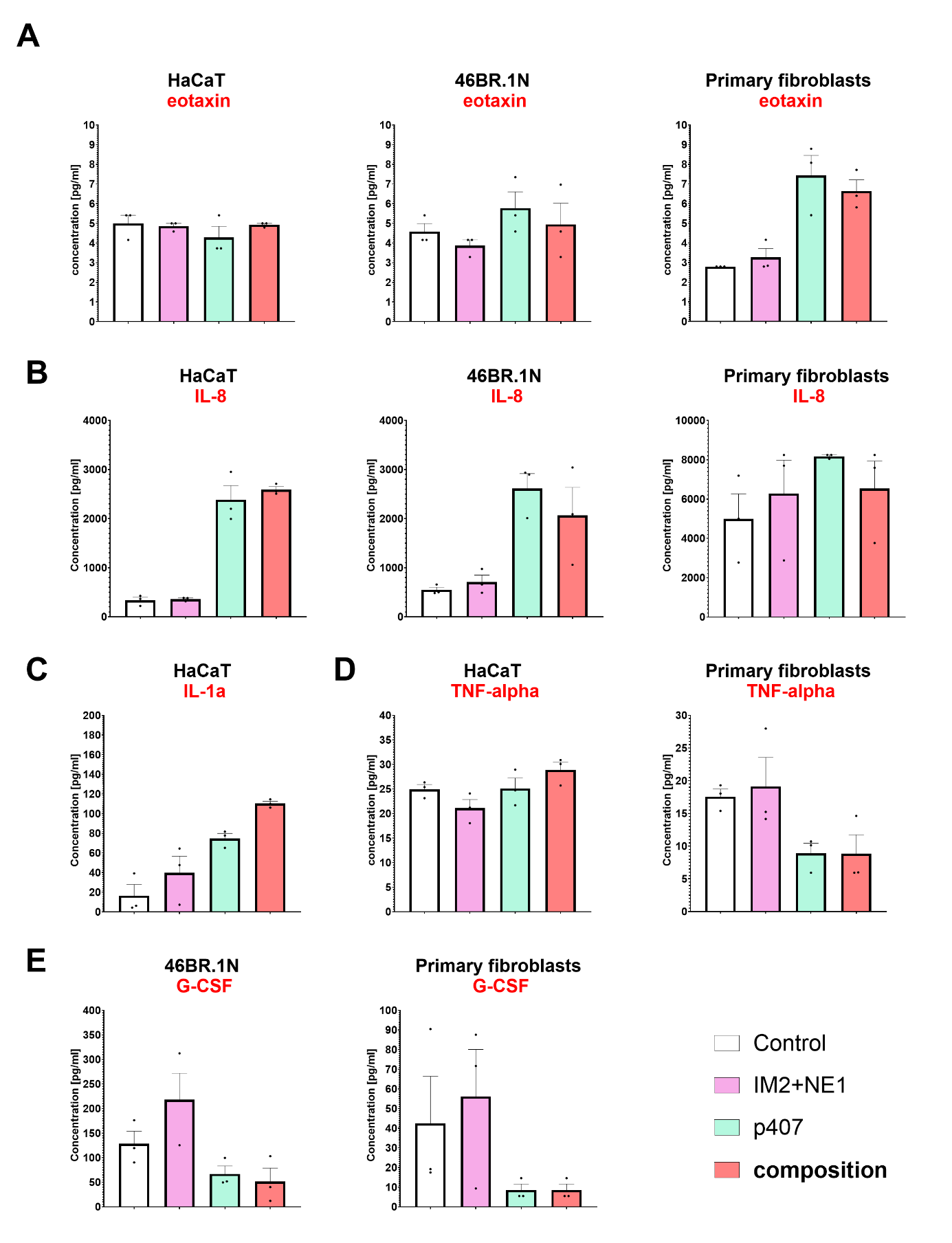


**Figure 1S**. **Levels of eotaxin (A), IL-8 (B), IL-1a (C), TNF-alpha (D), G-CSF (E) after IM2+NE1, P407 and composition stimulation of skin cells evaluated with Luminex technology.** Graphs present data from 3 independent experiments, no statistical significance was detected (Mann Whitney U-test, p<0.05).

Table 1S. Validation of the neutralization of the preservative system in the test using Eugon LT100

|  | | | | | | |
| --- | --- | --- | --- | --- | --- | --- |
| Tested stains | **Nvf**  (unit/ml*) | **Nvn**  (unit/ml) | **Nvf ≥ 0,5 Nvn*** | | **Nv**  (unit/ml) | |
| *S. aureus*  ATCC 6538 | 3,5 x 10^3^ | 5,2 x 10^3^ | | > 0,5 | | 5,6 x 10^3^ |
| *E. coli*  ATCC 8739 | 3,3 x 10^3^ | 4,9 x 10^3^ | | > 0,5 | | 5,3 x 10^3^ |
| *Ps. Aeruginosa*  ATCC 9027 | 3,0 x 10^3^ | 5,1 x 10^3^ | | > 0,5 | | 5,4 x 10^3^ |
| *C. albicans*  ATCC 10231 | 3,3 x 10^3^ | 5,3 x 10^3^ | | > 0,5 | | 5,7 x 10^3^ |
| *A. brasiliensis*  ATCC 16404 | 2,5 x 10^3^ | 3,4 x 10^3^ | | > 0,5 | | 3,7 x 10^3^ |

*unit/ml – colony forming unit in 1ml

** **Nvf ≥ 0.5 Nvn** – assumption according to PN-EN ISO 11930:2019

Nvf – number of microorganisms in 1ml of a mixture consisting of: 1ml of the test sample + 9ml of neutralizer + 1ml of the test strain suspension (N) from a dilution of 10^-4^ (bacteria)/10^-3^ (*C. albicans* and *A. brasiliensis*)

Nvn – Nvn – number of microorganisms in 1ml of a mixture of: 1ml of diluent + 9ml of neutralizer + 1ml of the test strain suspension (N) from a dilution of10^-4^ (bacteria)/10^-3^ (*C. albicans* and *A. brasiliensis*)

Nv – Nv – number of microorganisms in the control: 10 ml of diluent + 1 ml of suspension of the test strain (N) from a dilution of10^-4^ (bakteria)/10^-3^ (*C. albicans* and *A. brasiliensis*)

Table 2S. Challange test

| Density of suspensions of test strains used for contamination of the tested samples | | |
| --- | --- | --- |
| Test strains | **N** (unit/ml)  Number of microorganisms in a calibrated suspension | **N_0_ = N/100** (unit/ml)  The number of microorganisms in the sample tested immediately after contamination (T_0_) |
| *S. aureus* ATCC 6538 | 4,7 x 10^7^ | 4,7 x 10^5^ |
| *E. coli* ATCC 8739 | 4,2 x 10^7^ | 4,2 x 10^5^ |
| *Ps. Aeruginosa* ATCC 9027 | 4,0 x 10^7^ | 4,0 x 10^5^ |
| *C. albicans* ATCC 10231 | 3,6 x 10^6^ | 3,6 x 10^4^ |
| *A. brasiliensis* ATCC 16404 | 3,0 x 10^6^ | 3,0 x 10^4^ |

Table 3S. Number of microorganisms in 1 ml of sample at specified time intervals

| Test strains | T_0_  (unit/ml) | Ig N_0_ | | T_7_  (unit/ml) | Ig N_7_ | T_14_  (unit/ml) | Ig N_14_ | T_28_  (unit/ml) | Ig N_28_ |
| --- | --- | --- | --- | --- | --- | --- | --- | --- | --- |
| *S. aureus* ATCC 6538 | 4,7 x 10^5^ | | 5,67 | <10 | 1 | <10 | 1 | <10 | 1 |
| *E. coli* ATCC 8739 | 4,2 x 10^5^ | | 5,62 | <10 | 1 | <10 | 1 | <10 | 1 |
| *Ps. Aeruginosa* ATCC 9027 | 4,0 x 10^5^ | | 5,60 | <10 | 1 | <10 | 1 | <10 | 1 |
| *C. albicans* ATCC 10231 | 3,6 x 10^4^ | | 4,55 | <10 | 1 | <10 | 1 | <10 | 1 |
| *A. brasiliensis* ATCC 16404 | 3,0 x 10^4^ | | 4,47 | <10 | 1 | <10 | 1 | <10 | 1 |

Table 4S. The value of the reduction of the number of microorganisms in the sample at time intervals

R_x_ = IgN_0_ - IgN_x_

| Tested strains | T_7_ | T_14_ | T_28_ |
| --- | --- | --- | --- |
| *S. aureus* ATCC 6538 | 4,67 | 4,67 | 4,67 |
| *E. coli* ATCC 8739 | 4,62 | 4,62 | 4,62 |
| *Ps. Aeruginosa* ATCC 9027 | 4,60 | 4,60 | 4,60 |
| *C. albicans* ATCC 10231 | 3,55 | 3,55 | 3,55 |
| *A. brasiliensis* ATCC 16404 | 3,47 | 3,47 | 3,47 |
| Reduction values ​​(Rx) are expressed in logarithmic units | | | |

Table 5S. Assessment criteria for the maintenance effectiveness test according to the standard PN-EN ISO 11930:2019

|  | Bacteria | | | *C. albicans* | | | *A. brasiliensis* | |
| --- | --- | --- | --- | --- | --- | --- | --- | --- |
| Time | T_7_ | T_14_ | T_28_ | T_7_ | T_14_ | T_28_ | T_14_ | T_28_ |
| Criterion A | ≥3 | ≥3 NI^a^ | ≥3 NI | ≥1 | ≥1 NI | ≥1 NI | ≥0^b^ | ≥1 |
| Criterion B | not tested | ≥3 | ≥3 NI | not tested | ≥1 | ≥1 NI | ≥0 | ≥0 NI |

Reduction values ​​calculated from the formula: R_x_ = IgN_0_ - IgN_x_

^a^ NI – the number of microorganisms does not increase

^b^ R_x_ = 0 when IgN_0_ = IgN_x_

Table 6. Stability and packaging compatibility testing

| Parameter | Requirements | Results |
| --- | --- | --- |
| Character | Transparent, clear liquid, odorless | Compatible |
| pH | - | 5,5 |
| Mechanical contamination | does not contain | absent |
| Sample stability at -5°C  10 cycles:  1. Placing the sample at a reduced temperature (-5°C) for 24 hours  2. Taking the sample out for 8 hours – return to room temperature  3. Evaluation of any changes / mass destabilization / reactivity with packaging  4. Placing the sample at a reduced temperature for 16 hours until the next evaluation | No signs of mass destabilization, reactivity with packaging or other changes | No changes observed |
| Sample stability at room temperature +18°C/+20°C without light  10 cycles:  1. Assessment of possible changes / mass destabilization / reactivity with packaging every 24h | No signs of mass destabilization, reactivity with packaging or other changes | No changes observed |
| Stability at +38°C  10 cycles:  1. Placing the sample at an elevated temperature for 24 hours  2. Taking the sample out for 8 hours – return to room temperature  3. Evaluation of any changes / mass destabilization / reactivity with packaging  4. Placing the sample in specified temperature conditions for 16 hours until the next evaluation | No signs of mass destabilization, reactivity with packaging or other changes | No changes observed |
| Stability of the preservation system: | ISO 11930:2019 | Compatible |
| Compatibility of weight with packaging | complete | Compatible |
